# Proportion of New Onset of Anosmia and Its Diagnostic Significance in COVID-19

**DOI:** 10.7759/cureus.24983

**Published:** 2022-05-13

**Authors:** Ripu Daman Arora, Kota Sesha Brahma Sree Krishna Sasanka, Pugazhenthan Thangaraju, Sree Sudha Tanguturi Yella, Nitin M Nagarkar

**Affiliations:** 1 Otorhinolaryngology-Head and Neck Surgery (ENT-HNS), All India Institute of Medical Sciences, Raipur, IND; 2 Otorhinolaryngology-Head and Neck Surgery (ENT-HNS), All India Institute of Medical Sciences, Deoghar, IND; 3 Pharmacology and Therapeutics, All India Institute of Medical Sciences, Raipur, IND; 4 Pharmacology, All India Institute of Medical Sciences, Deoghar, IND

**Keywords:** covid-19, loss of smell, olfactory dysfunction, hyposmia, anosmia, cosanar (corana scale on anosmia aiims raipur) scale, isca-r (indian smell test in covid by aiims raipur)

## Abstract

Objective: This study aimed to know the proportion of new-onset of anosmia and to find its diagnostic significance in coronavirus disease 2019 (COVID-19) patients attending the hospital.

Study design and duration: The Indian smell test in COVID-19 by AIIMS Raipur (ISCA-R) was developed for evaluating olfaction in the Indian population. The olfactory function was assessed using the corona scale on anosmia AIIMS Raipur (COSANAr).

Results: Out of 256 patients, 171 were males and 85 were females. In the majority of the patients, 75 (29.29%), the COSANAr score “0” was higher on the day of admission compared to the score “3” on the day of discharge with 61 (23.82%) patients. There was no improvement in 134 (52.34%) patients with loss of smell at the time of discharge.

Conclusion: This study is a step forward in identifying anosmia by ISCA-R at the early stages of the COVID phase. The COSANAr is affordable for the Indian population. It is noticed that most of the patients have mild hyposmia at the time of discharge and anosmia at the admission time.

## Introduction

The severity and expeditious spread of severe acute respiratory syndrome coronavirus 2 (SARS-CoV-2) are accrediting researchers worldwide to obtain a large amount of clinical data regarding coronavirus disease 2019 (COVID-19) [1]. COVID-19 has turned into a global emergency in the short time of its outbreak due to a beta coronavirus called SARS-CoV-2 [2]. The disease is transmitted through droplets and aerosol from COVID-19-positive patients [3]. The virus enters the host alveoli by binding to the angiotensin-converting enzyme 2 (ACE2) receptor through their spike protein [4,5]. Primary inflammatory mediators like IL1, IL6, and tumor necrosis factor (TNF)-alpha) cause adverse scenarios of the disease [6]. The common symptoms in many populations are fever, cough, sore throat, fatigue, breathlessness, anosmia, headache, and myalgia [2,4]. COVID-19 in Europe observed a high incidence of olfactory dysfunction [7-10]. Importantly, loss of smell appears to be very common in the initial stages of the disease, and most of the time, it is the only clinical presentation in patients [7,8]. To interrupt the spread of infection, it is needed to identify and isolate the paucity of symptomatic patients. So far, the area of chemosensory disorders in COVID-19-affected patients has not been quantitatively and qualitatively evaluated in the evidence-based literature. Due to the sudden spread of disease, most studies rely on subjective self-evaluations of patients, reports of anamnestic data, or olfactory questionnaire completion, with no objective test or a direct medical examination [8-10]. Early warning markers of COVID-19 infection were anosmia and autonomic dysfunction [11,12]. There is no rationale that why some patients are prone to a loss of smell and the mechanism of loss of smell remains elusive. There is a paucity of evidence on the findings of anosmia from Asia, particularly India, which was the impetus for the design of this study. In this study, patients with confirmed mild to moderate SARS-CoV-2 infections were evaluated for the proportion, onset of altered smell, and recovery time.

## Materials and methods

The research was held with the approval of the Institutional Ethics Committee of All India Institute of Medical Sciences, Raipur, India (approval no.: AIIMSRPR/IEC/2021/734). A cross-sectional study was carried out on COVID-19-positive patients who were confirmed by reverse transcription-polymerase chain reaction (RT-PCR) and admitted to AIIMS, Raipur. Positively, patients who were willing to give consent and cooperate in the study were enrolled. The study duration was 28 days. A total of 256 people with RT-PCR COVID-positive who were admitted to the AIIMS, Raipur, between February 16 and May 15, 2021, were recruited for this study. Patients with assisted ventilation, neurological disorders, a history of surgery or radiotherapy in the oral and nasal cavities, preexisting manifestations of smell and taste alterations, a history of head trauma, allergic rhinitis, chronic rhinosinusitis, and those taking anticancer, anti-thyroid, or opiate medications were all excluded from the study.

Study objectives

The primary objective of this study was to know the proportion of new-onset of anosmia and to find out its diagnostic significance in COVID-19 patients attending AIIMS Raipur. The secondary objective was to find out the time duration in the recovery of sense of smell in treated COVID-19 cases.

Development of a test and its design

**Figure 1:**
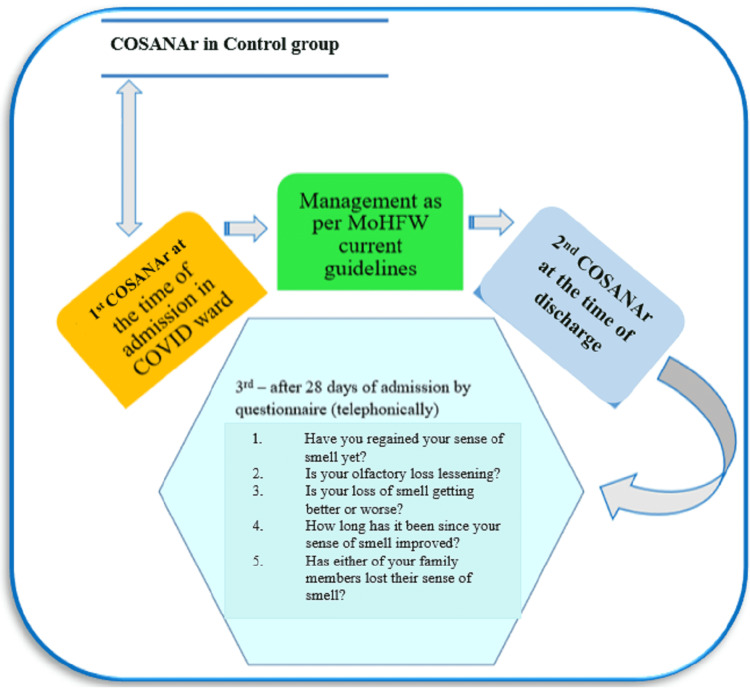
Flowchart of ISCA-R (Indian smell test in COVID-19 by AIIMS Raipur) MoHFW: Ministry of Health and Family Welfare; COSANAr: corona scale on anosmia AIIMS Raipur; COVID-19: coronavirus disease 2019

In the second wave of the pandemic, we attempted to develop a test such as the Indian smell test in COVID-19 by AIIMS Raipur (ISCA-R) to assess olfaction in the Indian population using limited resources (Figure 1). The test was conducted in three phases (the first and second phases were conducted objectively, while the third phase was conducted subjectively - telephonically). COVID-positive patients and COVID-negative patients (by RT-PCR) were compared for the first time at the time of admission, and both groups were of the same age/sex. A ratio of 2:1 (case:control) was used to select the cases and controls. The second time was at the time of discharge, and the third time was after 28 days of admission, using a prevalidated closed-end structured questionnaire over the phone (Figure 1). COVID-19-positive cases were managed in accordance with current Ministry of Health and Family Welfare (MoHFW) guidelines. The corona scale on anosmia AIIMS Raipur (COSANAr) will be used to assess olfactory function.

We standardized the two parameters (indigenous test and indigenous scale) through random and anonymous prevalidation, in accordance with the consent procedure. We chose the odors listed below. The indigenous scale scoring was done as per the COSANAr, as shown in the following. The sensitivity of the study will be increased if patients can identify the maximum number within the given sample.

Odors for Testing

The five odors listed below were chosen based on a pre-survey in normal healthy groups with proper consent (Table 1). These odors were obtained through the use of essences (liquid forms).

**Table 1:** Odors used in identifying loss of smell

S. No.	Odors
1	Camphor
2	Orange
3	Chocolate
4	Biryani
5	Jasmine

To determine the new onset of anosmia in COVID-19 patients, the function of loss of smell was graded using the COSANAr (Table 2). Based on the patient's response, a score of 1 or 0 was assigned for "yes or no." A final score was assigned, which is used to grade smell impairment.

**Table 2:** Grading of smell impairment by COSANAr scale COSANAr: corona scale on anosmia AIIMS Raipur

Grading of smell	Score
Normosmia	5
Normo hyposmia	4
Mild hyposmia	3
Moderate hyposmia	2
Severe hyposmia	1
Anosmia	0

Smell kit preparation steps

Test Kit Arrangement Phase

All of the sterile swab sticks were dipped in each odor for five minutes. Each fragranced swab stick was labeled with a number (1, 2, 3, 4, and 5). Each of the five numbered swab sticks was placed in its own container and packaged as a single pair kit. Each patient received a separate kit in order to determine their level of olfactory impairment.

Test Method

The patients were asked to pick up each swab stick, identify the smell, and mark the appropriate option on the answer sheet. The time interval between each smell identification is one minute. The smell grading function is based on the COSANAr. Each correct answer is worth one mark, while each incorrect answer is worth zero. The overall score is five. A score of five out of five (5/5) implies normosmia, a score of 4/5 implies normo hyposmia, a score of 3/5 implies mild hyposmia, a score of 2/5 implies moderate hyposmia, a score of 1/5 implies severe hyposmia, and a score of 0/5 implies anosmia.

Data analysis

All the information was exported to an Excel spreadsheet. We considered a p-value of 0.05 as statistically significant. As a result, the data gathered have been evaluated for definitive remarks. We employed the chi-square method for categorical data.

## Results

**Figure 2:**
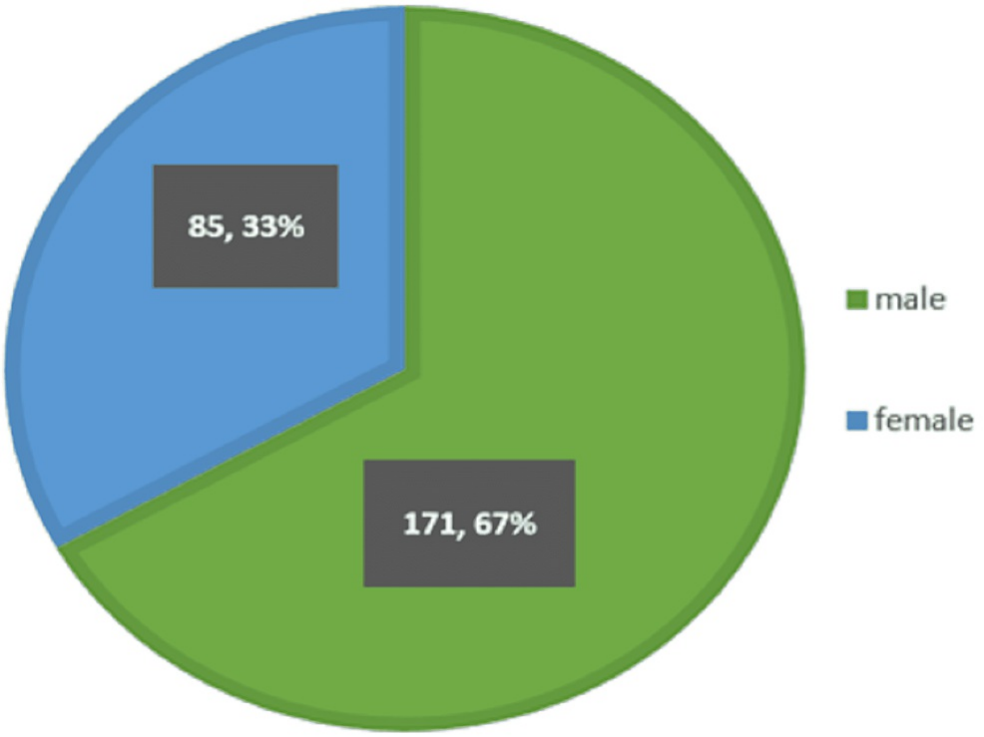
Representation of sex distribution affected by the loss of smell

**Figure 3:**
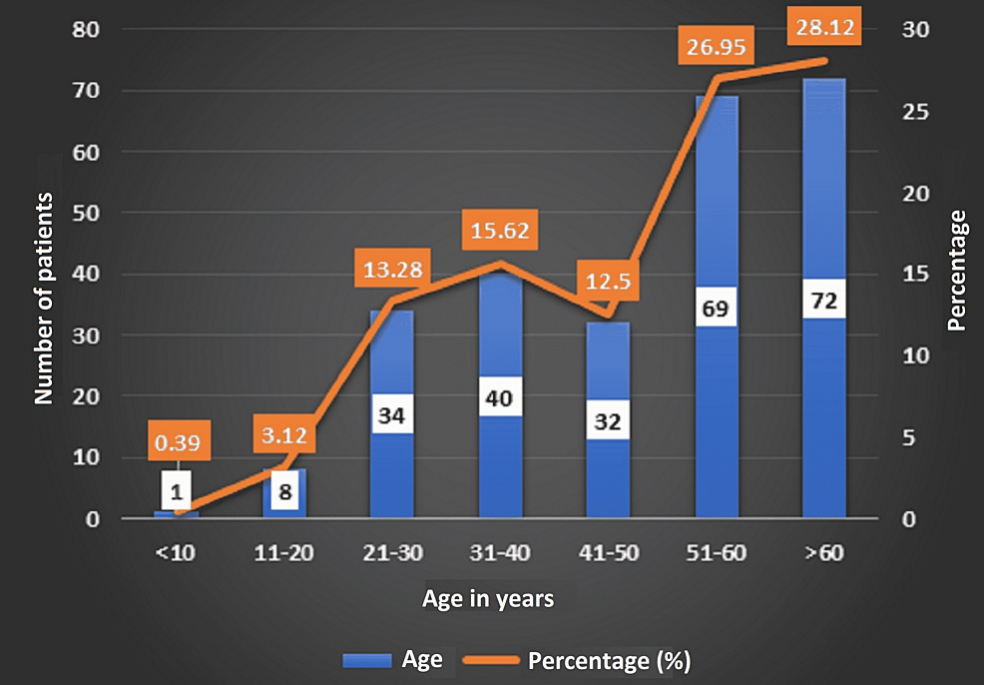
Age group wise distribution of the patients

In our study, 256 people with RT-PCR COVID-positive reports were admitted to the AIIMS, Raipur, between February 16 and May 15, 2021. There was contact information for 300 people, 44 of whom were excluded based on criteria. Males were more affected than females (Figure 2). In our study, the majority of the patients were over the age of 51 years (Figure 3).

We tested the patients objectively twice (during admission and discharge) with the COSANAr. The findings are summarized in Table 3. In our study, the majority of the patients (75 {29.29%}) had anosmia at the time of admission, and 61 (23.82%) had mild hyposmia on the day of discharge, with a significant p-value of <0.05 (Table 3).

**Table 3:** Objective findings, anosmia score, by using COSANAr in COVID-19 patients *Significant value. COSANAr: Corana Scale on Anosmia AIIMS Raipur; COVID-19: coronavirus disease 2019

COSANAr score	On the day of admission	On the day of discharge	p-Value
	No. of patients	No. of patients	
0, anosmia	75	35	0.000017^*^
1, severe hyposmia	21	22	0.873404
2, moderate hyposmia	48	50	0.822233
3, mild hyposmia	41	61	0.026901^*^
4, normo hyposmia	32	50	0.03008^*^
5, Normosmia	39	38	0.901603

**Figure 4:**
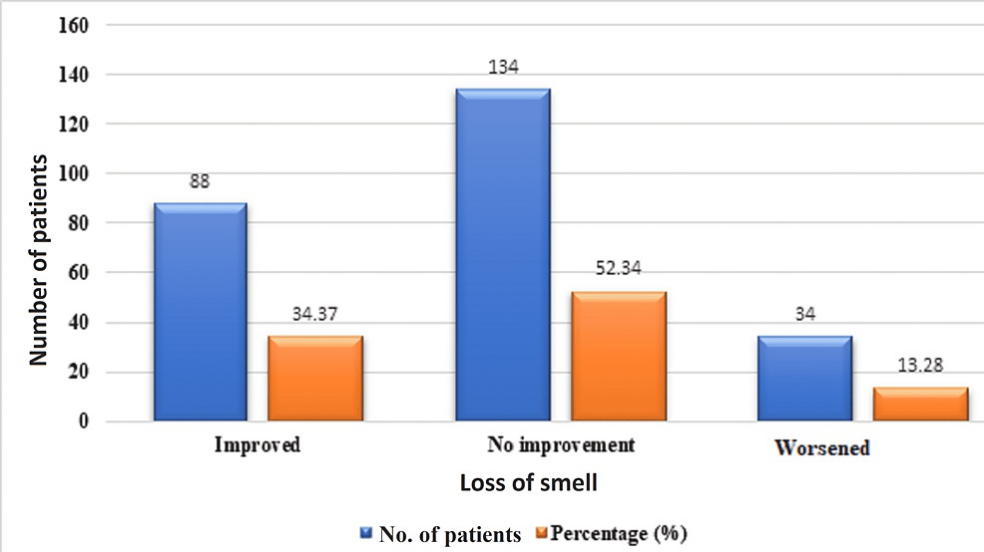
Comparison of loss of smell between at the time of admission and discharge

The patients in Figure 4 have been classified based on a comparison of both objective COSANAr scores. When the initial score is lower/poor (loss of smell present) and the subsequent score is higher/improved (recovering/improving loss of smell), it is stated as "improved." When there is no change in score across both tests, it is referred to as "no improvement." It is referred to as worsened when the score is higher/good (during admission) and poor/lower (while discharge).

According to the ISCA-R test, the third phase should be conducted subjectively by a questionnaire over the phone on the 29th day after admission. It has been found that nearly three-quarters of patients recover in more than 14, 20, and even 28 days. It was noticed in the follow-up that no family members had been affected by the loss of smell. As shown in Table 4, a set of questions has been advanced for the follow-up.

**Table 4:** Subjective findings, loss of smell, during the follow-up (telephonically)

Questions	Reply (no. of patients)
Still, you have loss of smell?	No (207); yes (11)
Is your loss of smell improved partially/completely?	Completely (182); partially (25); no improvement (11)
Since how many days your loss of smell improved?	Less than 14 days (107); more than 14 days (111)

According to the ISCA-R, the control group must also be tested. The total number of controls in our study is 128. The findings are summarized in Table 5.

**Table 5:** Objective findings, anosmia score, by using COSANAr and subjective findings during follow-up (telephonically) in the control group COSANAr: corona scale on anosmia AIIMS Raipur

COSANAr score	On the 1st day	On the 5th day	On the 28th day
	Controls	Controls	Controls
4, normo hyposmia	09	4	1
5, normosmia	119	124	127

## Discussion

Complete anosmia was observed in 75 (29.29%) patients in our study, whereas complete anosmia was detected only in 2.8% of patients in Vaira et al.'s objective evaluations, with most patients presenting with varying degrees of hyposmia (80.6%) [13]. In our study, 55.46% and 16.7% of patients had normal olfactory function, respectively; however, 39 patients were found to have normal smell function (15.23%).

Initially, anosmia was not assumed to be a significant symptom of coronavirus disease. From the literature, several reports of anosmia presented as a common symptom. Anosmia was added to the list of common manifestations by the American Academy of Otolaryngology-Head and Neck Surgery and the British Association of Otorhinolaryngology, who also recommended screening to detect anosmia [14]. It is still difficult to detect coronavirus carriers non-invasively. Despite the fact that anosmia is a rare illness, it appears to be a promising marker of the disease. The cause of olfactory dysfunction in COVID-19 is unclear. Rhinovirus, parainfluenza virus, coronavirus, and Epstein-Barr virus are the most commonly associated viruses with such dysfunction [14].

We found that olfactory dysfunction, anosmia, is a common symptom of COVID-19 in this study. which is reported by a significant proportion of 29.29% at the time of admission in the patients. Still, 23.82% of patients had mild hyposmia on the day of discharge. We have proved through our newly developed ISCA-R and COSANAr that olfaction is compromised in COVID patients. As all the patients were followed up telephonically on the 29th day and maximum in the first 28 days, we noticed an 87% recovery rate in smell disturbances in our study as compared to another research reported 59.7% incidences of no recovery rate in smell disturbances [15].

Moein et al. assessed odor function in 60 COVID patients using the University of Pennsylvania Smell Identification Test (UPSIT) method and found smell dysfunction in 98% of them, with 25% anosmic, whereas in our study the anosmic percentage is slightly higher on the day of admission and even higher (33%) on the day of discharge, with 33% severely hyposmic, 27% moderately hyposmic, 13% mildly hyposmic, and 2% normosmic [16].

The current study takes a step forward in identifying anosmia in asymptomatic COVID-19 patients by incorporating five readily available odors. To the best of our knowledge, this is the first study in India that is not solely focused on questionnaire surveys or telephonic interviews, and in which five different odors were used, to which, the patients answered back and filled in their responses. This is the only study in which both objective and subjective findings are documented. Bhattacharjee et al. used a novel olfactometer to measure the severity of anosmia and reported that only 15% of the 85% of anosmic asymptomatic patients in their study were aware of their loss of smell [17]. In the preceding study, asymptomatic cases were difficult to identify, and in such scenarios in low-income countries with a large population, this cost-effective test allows us to screen each individual in India, which is otherwise not possible through any other form of testing. Overall, the current study provides preliminary data indicating a loss in smell sensation for various odors.

Limitations

It was a one-center study. In addition, patients' follow-up time was restricted to one month only. To establish a strong relationship, larger sample size is required. To compare the sensitivity and specificity of our method, we used new methodology but not the gold standard test.

## Conclusions

Anosmia can be a sign of coronavirus infection. ISCA-R olfaction test can be useful for easy and earlier detection of anosmia at a minimal price in low-income countries with a large population, and it can be used reliably in all patients in the period of the pandemic.
